# Conversion of a Non-Cancer-Selective Promoter into a Cancer-Selective Promoter

**DOI:** 10.3390/cancers14061497

**Published:** 2022-03-15

**Authors:** Praveen Bhoopathi, Anjan K. Pradhan, Amit Kumar, Santanu Maji, Padmanabhan Mannangatti, Xiaoyan Deng, Dipankar Bandyopadhyay, Devanand Sarkar, Xiang-Yang Wang, Joseph W. Landry, Swadesh K. Das, Luni Emdad, Paul B. Fisher

**Affiliations:** 1 Department of Human and Molecular Genetics, School of Medicine, Virginia Commonwealth University, Richmond, VA 23298, USA; praveen.bhoopathi@vcuhealth.org (P.B.); anjan.pradhan@vcuhealth.org (A.K.P.); amit.kumar@vcuhealth.org (A.K.); santanu.maji@vcuhealth.org (S.M.); padmanabhan.mannangatti@vcuhealth.org (P.M.); devanand.sarkar@vcuhealth.org (D.S.); xiang-yang.wang@vcuhealth.org (X.-Y.W.); joseph.landry@vcuhealth.org (J.W.L.); swadesh.das@vcuhealth.org (S.K.D.); 2 VCU Institute of Molecular Medicine, School of Medicine, Virginia Commonwealth University, Richmond, VA 23298, USA; 3 Department of Biostatistics, School of Medicine, Virginia Commonwealth University, Richmond, VA 23298, USA; xiaoyan.deng@vcuhealth.org (X.D.); bandyop@vcuhealth.org (D.B.); 4 VCU Massey Cancer Center, School of Medicine, Virginia Commonwealth University, Richmond, VA 23298, USA

**Keywords:** transcriptional regulation, cancer selective promoter, PEG-3, GADD34, GATA2, tumor and metastasis imaging

## Abstract

**Simple Summary:**

The rat progression elevated gene-3 (PEG-3) promoter displays cancer-selective expression, whereas the rat growth arrest and DNA damage inducible gene-34 (GADD34) promoter lacks cancer specificity. PEG-3 and GADD34 minimal promoters display strong sequence homology except for two single point mutations. Since mutations are prevalent in many gene promoters resulting in significant alterations in promoter specificity and activity, we have explored the relevance of these two nucleotide alterations in determining cancer-selective gene expression. We demonstrate that these two point mutations are required to transform a non-cancer-specific promoter (pGADD) into a cancer-selective promoter (pGAPE). Additionally, we found GATA2 transcription factor binding sites in the GAPE-Prom, which regulates pGAPE activity selectively in cancer cells. This newly created pGAPE has all the necessary elements making it an appropriate genetic tool to noninvasively deliver imaging agents to follow tumor growth and progression to metastasis and for generating conditionally replicating adenoviruses that can express and deliver their payload exclusively in cancer.

**Abstract:**

Progression-elevated gene-3 (PEG-3) and rat growth arrest and DNA damage-inducible gene-34 (GADD34) display significant sequence homology with regulation predominantly transcriptional. The rat full-length (FL) and minimal (min) PEG-3 promoter display cancer-selective expression in rodent and human tumors, allowing for cancer-directed regulation of transgenes, viral replication and in vivo imaging of tumors and metastases in animals, whereas the FL- and min-GADD34-Prom lack cancer specificity. Min-PEG-Prom and min-GADD34-Prom have identical sequences except for two single-point mutation differences (at −260 bp and +159 bp). Engineering double mutations in the min-GADD34-Prom produce the GAPE-Prom. Changing one base pair (+159) or both point mutations in the min-GADD34-Prom, but not the FL-GADD34-Prom, results in cancer-selective transgene expression in diverse cancer cells (including prostate, breast, pancreatic and neuroblastoma) vs. normal counterparts. Additionally, we identified a GATA2 transcription factor binding site, promoting cancer specificity when both min-PEG-Prom mutations are present in the GAPE-Prom. Taken together, introducing specific point mutations in a rat min-GADD34-Prom converts this non-cancer-specific promoter into a cancer-selective promoter, and the addition of GATA2 with existing AP1 and PEA3 transcription factors enhances further cancer-selective activity of the GAPE-Prom. The GAPE-Prom provides a genetic tool to specifically regulate transgene expression in cancer cells.

## 1. Introduction

Defining the steps necessary to convert a normal cell into a cancerous one has been aided by identification and interrogation of defined genetic elements that regulate these processes [1,2,3]. Using adenovirus-transformed primary rat embryo cells combined with subtraction hybridization [4,5], unique cancer-promoting progression-elevated genes (PEG) have been identified [5,6]. This strategy resulted in the identification and cloning of the progression-elevated gene-3 (PEG-3) gene [5], which displays enhanced expression in adenoviral-transformed rat cells expressing diverse oncogenes [5]. This same PEG-3 gene induces transformed/tumorigenic phenotypes in vivo in animal models [4,5]. Forced overexpression of PEG-3 enhances anchorage-independent growth and increased tumorigenicity, while knocking down PEG-3 expression inhibits tumorigenesis in nude mice [5,6].

The N-terminal domain (first 415 aa) of rat PEG-3 is identical to the rat GADD34 protein [5,7,8]. The *peg-3* and *gadd34* genes share 73% nucleotide and 59% amino acid sequence homology [5]. The *peg-3* and the murine *gadd34* genes [5,9] have extensive sequence homology (68% nucleotide and 72% aa similarities) [5]. In contrast, the carboxyl terminus of PEG-3 differs significantly from GADD34 with only 28% and 40% homology in the carboxyl-terminal 88 aa. These differences in the carboxyl terminus of PEG-3 vs. GADD34 proteins offer a plausible explanation for the functional differences between these genes [5,9]. Overexpression of GADD34 inhibits growth and induces apoptosis [7,10,11,12,13]. GADD34 overexpression leads to p53 phosphorylation and upregulates p21 proteins, which might be the underlying cause of GADD34-mediated apoptosis and growth arrest [8,14]. In contrast, the overexpression of PEG-3 in rodent or human tumor cells results in aggressive tumorigenic properties by inducing genomic instability, enhanced cellular invasion and increased tumor angiogenesis [6]. These studies indicate that, despite their sequence homology, GADD34 is a growth-suppressing and apoptosis-inducing gene, whereas PEG-3 facilitates tumor progression.

Previous studies indicate that regulation of both the *peg-3* and *gadd34* genes is chiefly transcriptional [5]. The full-length PEG-3 promoter is active in both human and rodent tumors, with minimal expression in normal cells [15,16]. Promoter deletion assays identified a minimum region of the PEG-3 promoter (−118 to +194) that was sufficient for its enhanced activity associated with transformation and cancer progression [9,15,16]. Sequence analysis, followed by gel shift and EMSA, revealed that this minimal promoter contains functional PEA-3 (+104) and AP-1 (+8) elements [9]. Further mutational analysis and direct transfection and functional assays verified that both PEA-3 and AP-1 are critical regulators of the promoters’ activity, both for de novo (basal) and oncogene induced [9,15,16].

Mutations are common events in gene promoters, which can result in dramatic changes in promoter specificity and activity [17,18,19,20,21]. Our previous work discovered that the minimal rodent PEG-3 promoter has high sequence similarity to the rat GADD34 promoter [5]. More specifically, this minimal PEG-3 promoter sequence has only a two base pair difference with GADD34, giving them 99% identity. In the present study, this sequence difference was investigated experimentally to understand the significance of these two nucleotide changes in defining cancer-selective gene expression. By further studying the cancer-specific mechanisms of the PEG-3 promoter, we will validate its multiple uses in regulating expression of transgenes selectively in cancer.

## 2. Results

### 2.1. Conversion of the Rat Min-GADD34-Prom (pGADD) into a Cancer-Selective Promoter (pGAPE)

**Figure 1:**
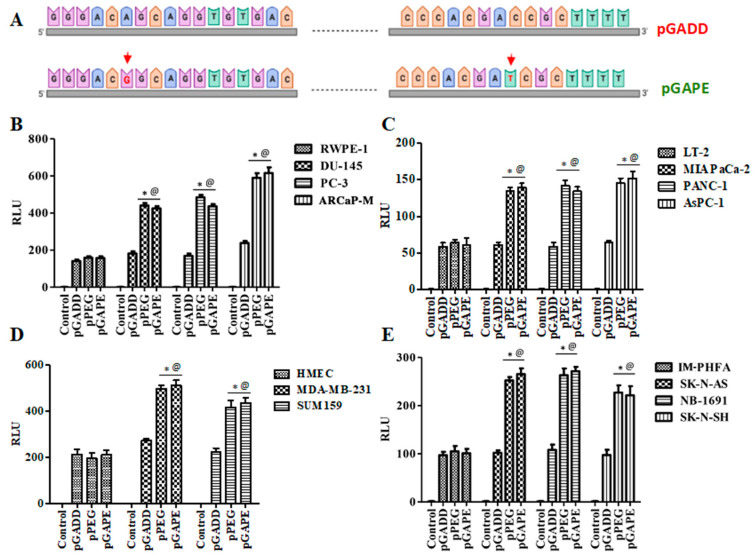
pGAPE displays similar elevated expression in cancer cells, as does the pPEG. (**A**) Schematic representation of conversion of pGADD to pGAPE. (**B**) Immortalized human prostate epithelial (RWPE-1) and prostate cancer (DU-145, PC-3 and ARCaP-M) cells. (**C**) Human immortalized pancreatic mesenchymal (LT-2) and pancreatic cancer (MIA PaCa-2, AsPC-1 and PANC-1) cells. (**D**) Primary human mammary epithelial (HMEC) and breast cancer (MDA-MB-231 and SUM159) cells. (**E**) Immortalized primary human fetal astrocytes (IM-PHFA) and neuroblastoma (SK-N-AS, NB-1691 and SK-N-SH) cells were transfected with the PGL4-Luc (Control), rat min-GADD34-Prom (pGADD), min-PEG-Prom (pPEG) or GAPE-Prom (pGAPE) for 48 h. Expression was normalized using pRL-TK, and the luminescence readings were plotted as relative luminescence units (RLU). The results presented are from three independent experiments with triplicate samples for each experimental variable. *, *p* < 0.01 vs. pGADD in each cell line (FDR corrected); @, *p* < 0.01 vs. pPEG/pGAPE (FDR corrected) in normal primary or immortal cell line.

To determine why the cancer-specific min-PEG-Prom (pPEG) is functionally different than the pGADD, which shares almost identical sequence similarity, we compared the sequence of the promoter regions of the pPEG (465 bp, from −270 to +195) with the pGADD (465 bp, from −281 to +184), which resulted in the identification of only two base pairs that were different (Supplementary Figure S1). Using standard PCR primers and site-directed mutagenesis, we first mutated these two base pairs in the pGADD, such that it was converted into pPEG, hereafter called pGAPE (Figure 1A, Supplementary Figure S1). These engineered minimal pPEG/pGADD/pGAPE promoters were cloned into the pGL4.14 vector and were transfected into human cells (cancer and primary/immortal normal) along with pRL-TK (Renilla, as a control), and luminescence was measured using the Dual-Glo luciferase (Luc) assay system.

### 2.2. Comparison of Cancer Specificity of the pPEG, pGADD and pGAPE

Using the luciferase assay, we compared the activity of the pGADD-, pPEG- and pGAPE-Luc in cancer vs. normal (immortalized or primary) human cells. As shown in Figure 1B, the relative activity of pPEG and pGAPE was identical and significantly higher in human prostate cancer cells (~3–4 fold) in comparison with normal human RWPE-1 prostate epithelial cells, whereas pGADD activity lacked cancer-selective activity and was similar in prostate cancer and RWPE-1 cells. Similarly, the activity of pPEG and pGAPE was ~3–4-fold higher in human pancreatic cancer cell lines in comparison with normal immortal LT-2 human pancreatic cells (Figure 1C). Breast cancer and neuroblastoma cells showed a ~2–3-fold increase in activity of pPEG and pGAPE vs. HMEC (early passage normal primary human breast epithelial cells) or IM-PHFA (immortal primary human fetal astrocytes) (Figure 1D,E). In all the cell lines, pGADD activity was not significantly changed between human cancer and respective immortalized or primary normal cells. These findings confirm that by simply reverting the two point mutations in the pGADD, a reporter construct that is devoid of cancer selectivity was converted into a cancer-specific promoter (pGAPE), which showed identical enhanced cancer-selective activity, as does pPEG.

### 2.3. Cancer Specificity of pPEG and pGAPE Promoters In Vivo in Tumor-Bearing Animal

**Figure 2:**
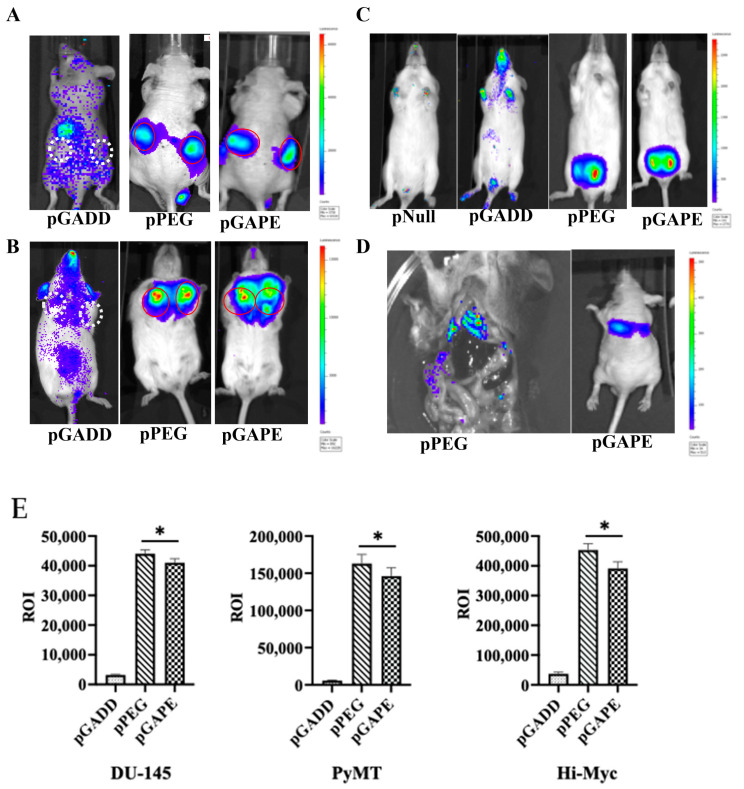
Tumor specificity of pGAPE. (**A**) DU-145 tumor xenografts in male athymic nude mice (*n* = 5) were intravenously injected with a Luc expression construct pGADD-Luc-PEI polyplex, pPEG-Luc-PEI polyplex or pGAPE-Luc-PEI polyplex, and BLI was performed after 48 h by IVIS. pGADD tumors negative for imaging are shown with white broken circles, and pPEG and pGAPE tumors positive for imaging are shown with solid red circles. (**B**) Tumor-bearing transgenic PyMT (*n* = 5) mice were treated as above, and BLI was performed after 48 h by IVIS. pGADD tumors negative for imaging are shown with white broken circles, and pPEG and pGAPE tumors positive for imaging are shown with solid red circles. (**C**) Tumor-bearing transgenic Hi-Myc mice (*n* = 5) were treated as above, and BLI was performed after 48 h by IVIS. We used control vector without the luciferase gene in the pNull group. (**D**) 4T1 breast cancer cells were intravenously injected into immune-competent mice (Left panel), and PC3-ML cells were intravenously injected into nude mice (right panel) followed by intravenous injection of pPEG-Luc-PEI polyplex or pGAPE-Luc-PEI polyplex, respectively. BLI was performed after 48 h by IVIS. (**E**) ROI determination for different tumor models. (Left panel) Total ROI calculated from DU-145 tumor-bearing mice (from **A**) and represented in graphical manner. (Center panel) Total ROI is calculated from PyMT (from **B**) and represented in graphical manner. (right) Total ROI is calculated from Hi-Myc mice and represented in graphical manner. *, *p* < 0.01 vs. pGADD.

Previous studies have confirmed that when pPEG-Luc or pPEG-HSV-Tk were complexed with in vivo jetPEI a linear polyethylenimine (l-PEI), intravenous injection permitted imaging of both primary tumors and metastases [22]. We initially confirmed similar cancer-specificity of pGAPE, which, based on its sequence, should show identical tumor imaging properties in vivo in animal models, using human DU-145 prostate cancer cell subcutaneous xenografts in nude mice (Figure 2A). Additionally, we examined two genetically engineered syngeneic mouse models, one developing breast and the other prostate cancer, which develop tumors spontaneously when specific transgenes are expressed in breast (PyMT) [23,24] or prostate (c-Myc) [25,26] under control of a mammary (MMTV)- or prostate (probasin)-specific promoter, respectively (Figure 2B,C). We also evaluated activity in experimental metastatic breast and prostate cancer mouse models, which develop lung metastases following intracardiac injections of murine 4T1 or human PC3-ML cells (Figure 2D). As an imaging component, we used Luc, frequently employed for bioluminescence imaging (BLI), to establish proof-of-principle for imaging specific gene expression or gene-tagged cells in preclinical animal models.

We used in vivo jetPEI for delivery and imaging of the different promoters [22,27]. JetPEI was used rather than a viral delivery system to avoid biased systemic delivery, as observed with certain viral vectors, which tend to localize to liver upon intravenous administration. After confirmation of the presence of palpable tumors in subcutaneous DU-145 xenografts in nude mice (Figure 2A) and in transgenic breast cancer PyMT mice (Figure 2B), an intravenous dose of pPEG-Luc, pGAPE-Luc or pGADD-Luc-PEI polyplex was administered by I.V. Forty-eight hours after plasmid DNA delivery, luciferin was administered, and mice were evaluated for pPEG-Luc, pGAPE-Luc and pGADD34-Luc gene expression by BLI. We used the same plasmid DNA delivery and imaging protocols in a group of healthy mice as negative controls. As shown in Figure 2, in all the experimental preclinical models, luciferase could be detected by BLI specifically in the tumors when driven by pPEG or pGAPE, whereas luciferase activity driven by pGADD was not specific to the tumors. Additionally, lung metastases that developed following intracardiac administration of 4T1 (Figure 2D, left) or PC3-ML (Figure 2D, right) cells were detected by BLI following injection with pPEG-Luc-PEI and pGAPE-Luc-PEI. Using different tumor animal models, pGAPE-Luc-PEI-injected animals showed similar cancer-specific BLI levels compared to pPEG-Luc-PEI-injected animals (Figure 2E). Control mice demonstrated nearly background levels of BLI output.

### 2.4. Maximum Conversion of the Non-Cancer-Selective pGADD to a Cancer-Selective pGAPE Requires Both Mutations Found in pPEG

**Figure 3:**
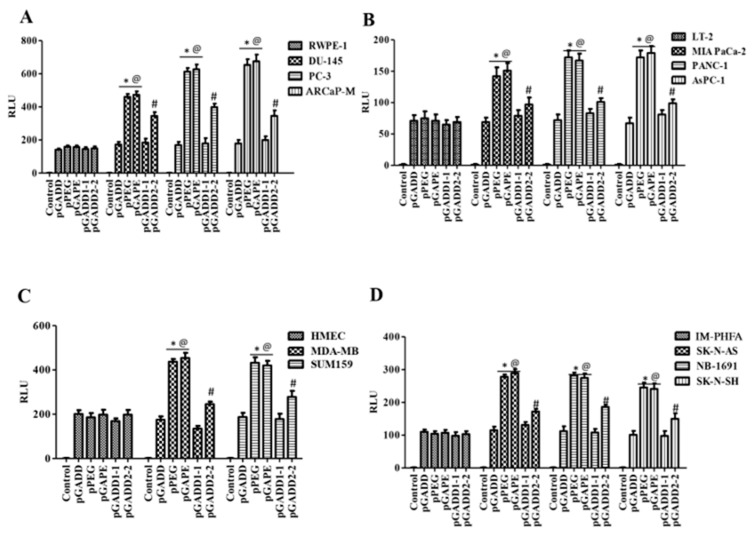
A single C-T mutation in the pGADD results in cancer specificity, which is less active than pGADD with two mutations. (**A**) Immortalized human prostate epithelial (RWPE-1) and prostate cancer (DU-145, PC-3 and ARCaP-M) cells. (**B**) Human immortalized pancreatic mesenchymal (LT-2) and pancreatic cancer (MIA PaCa-2, AsPC-1 and PANC-1) cells. (**C**) Primary human mammary epithelial (HMEC) and breast cancer (MDA-MB-231 and SUM159) cells. (**D**) Immortalized primary human fetal astrocytes (IM-PHFA) and neuroblastoma (SK-N-AS, NB-1691 and SK-N-SH) cells were transfected with PGL4-Luc (control), pGADD, pPEG, pGAPE, pGADD1-1 or pGADD2-2 for 48 h. Expression was normalized using pRL-TK, and the luminescence readings were plotted as relative luminescence units (RLU). The results presented are from three independent experiments with three replicates per experimental condition. *, *p* < 0.01 vs. pGADD (FDR corrected) within the individual cell lines; #, *p* < 0.05 vs. pGADD (FDR corrected) within the individual cell line; @, *p* < 0.01 vs. pPEG/pGAPE (FDR corrected) in a normal primary or immortalized normal cell line.

An important question was whether a single mutation in the pGADD was sufficient, or if both mutations were necessary, for pGAPE to display cancer-specific activity. To accomplish this goal, we generated a pGADD with a single mutation at bp −260 (G-A, termed pGADD1-1) and a second pGADD mutation at bp +159 (C-T, termed pGADD2-2). Using the same in vitro Luc-assay-based approach (described above), we analyzed the activity of pGADD, pPEG, pGAPE, pGADD1-1 and pGADD2-2 in the same series of human cancer, human immortalized normal cell lines, and primary normal cells shown in Figure 1. The relative activity of pPEG and pGAPE was ~3–4 fold higher in prostate cancer cells in comparison with normal human immortalized prostate epithelial cells (RWPE-1) (Figure 3). Although not as active as pPEG or pGAPE (with both mutations), the activity of pGADD2-2 (mutation at bp +159) was significantly higher in prostate cancer cells as compared to RWPE-1 (Figure 3A). In contrast, pGADD and pGADD1-1 activity in cancer cells was similar in prostate cancer cells and in RWPE-1. Similar effects as observed in prostate cancer cells were evident in pancreatic cancer, breast cancer and neuroblastoma cells (Figure 3B–D). In all the cell types tested, pGADD and pGADD1-1 activity was not significantly changed between cancer and respective immortalized/primary normal cells. Since pGADD2-2 activity was higher in cancer cells compared to respective normal cells in vitro, we evaluated the specificity of this mutant promoter for tumor imaging in the PyMT breast cancer animal model. After confirmation of palpable tumors in PyMT mice, the mice received an I.V. dose of pGADD1-1-Luc-PEI polyplex or pGADD2-2-Luc-PEI polyplex. Forty-eight hours after plasmid DNA delivery, promoter–driven gene expression was monitored by BLI (Supplementary Figure S2). The promoter with a single mutation at bp −260 lacked tumor-specific imaging ability in the PyMT model, whereas a single mutation at bp +159 in pGADD (minGADD-34-Prom) displayed cancer selectivity (which was less sensitive than observed in pGAPE containing both pPEG mutations). Taken together, these results suggest that reduced in vitro and in vivo cancer selectivity is retained, albeit to a lesser extent when a single mutation is engineered in bp +159 (pGADD2-2), whereas cancer selectivity and tumor-specific imaging is absent in pGADD1-1 (with a mutation only in bp −260).

### 2.5. Identification of an Additional Transcription Factor, GATA2, Promoting Cancer-Specific Activity of pPEG and pGAPE

**Figure 4:**
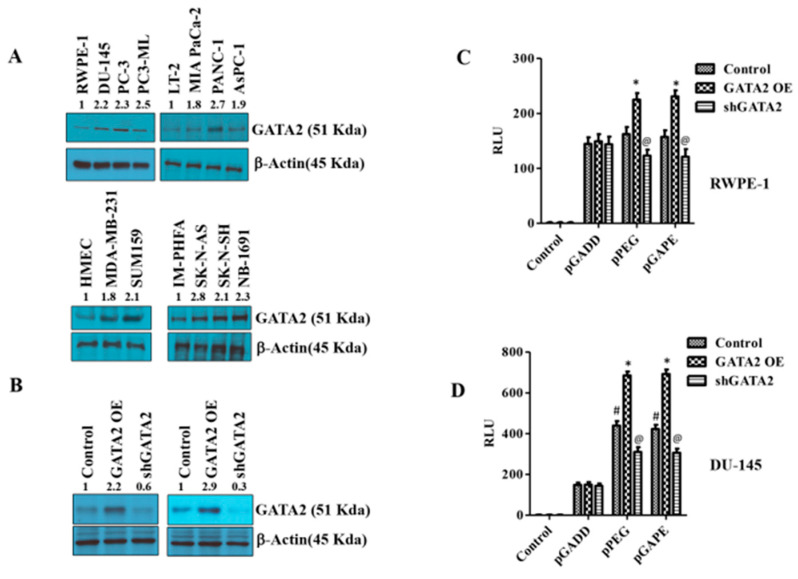
Changes in GATA2 expression affect cancer specificity. (**A**) Western blotting analysis for GATA2 expression levels in different cancer cells (prostate, pancreas, breast and neuroblastoma) compared with immortalized or primary normal cells. (**B**) RWPE-1/DU-145 cells were either transfected with a GATA2 overexpression (OE) plasmid or with an shGATA2 (small hairpin inhibitory RNA) plasmid, then were cultured for 48 h, and cells were collected, lysed and used for Western blotting. Blots were stained for GATA2, or β-Actin as a loading control. (**C**) RWPE-1 and (**D**) DU-145 cells were either transfected with a GATA2 OE plasmid or with a shGATA2 plasmid, cultured for 48 h and transfected with PGL4-Luc (control), pGADD, pPEG or pGAPE for an additional 48 h. Expression was normalized using pRL-TK, and the luminescence readings were plotted as relative luminescence units (RLU). The results presented are from three independent experiments. #, *p* < 0.01 vs. pGADD; *, *p* < 0.01 vs. pGADD-GATA2 OE; @, *p* < 0.05 vs. pPEG/pGAPE control (FDR corrected).

pPEG and pGAPE show tumor-specific in vivo imaging capabilities as well as enhanced tumor-specific expression in vitro, whereas the full-length GADD promoter, pT-GADD, does not show tumor specificity compared to normal cells. The same is true with the single mutation modification of bp −260 (pGADD1-1), which lacks cancer specificity, whereas the change of bp +159 (pGADD2-2), shows cancer selectivity when engineered in pGADD (the minimum GADD34-Prom). To check the transcription factors involved in this cancer-selective activity, we analyzed the pGADD with and without mutations with the TFBIND software [28], which predicts potential transcription factors. TFBIND software predicted GATA2 as an important transcription factor involved in defining cancer specificity when both mutations were present in the pGADD (Supplementary Figure S3). In order to compare GATA2 expression in cancers, we determined GATA2 protein levels in different cancer cell lines by Western blotting. GATA2 was significantly increased in carcinomas and neuroblastomas compared to respective normal cells (Figure 4A, Supplementary Figure S4A). Considering these results, we hypothesized that GATA2 might directly or indirectly regulate pGAPE/pPEG expression levels in cancer cells. To clarify the regulatory role of GATA2 on pGAPE/pPEG activity, we overexpressed (OE) or knocked down (KD) GATA2 in RWPE-1 and DU-145 cells, and the efficiency of OE or KD was confirmed by Western blotting (Figure 4B, Supplementary Figure S4B). We evaluated the levels of pPEG, pGADD and pGAPE activity in DU-145 and RWPE-1 cells in the GATA2-overexpressed and GATA2-downregulated cells using dual luciferase assays. Overexpressing GATA2 enhanced pGAPE and pPEG activity in both RWPE-1 and DU-145 cells, whereas pGADD activity was not altered. Similarly, GATA2 inhibition reduced the activity of pGAPE and pPEG but did not change pGADD activity in either cell line (Figure 4C,D).

### 2.6. GATA2 Directly Binds to pGAPE and pPEG

**Figure 5:**
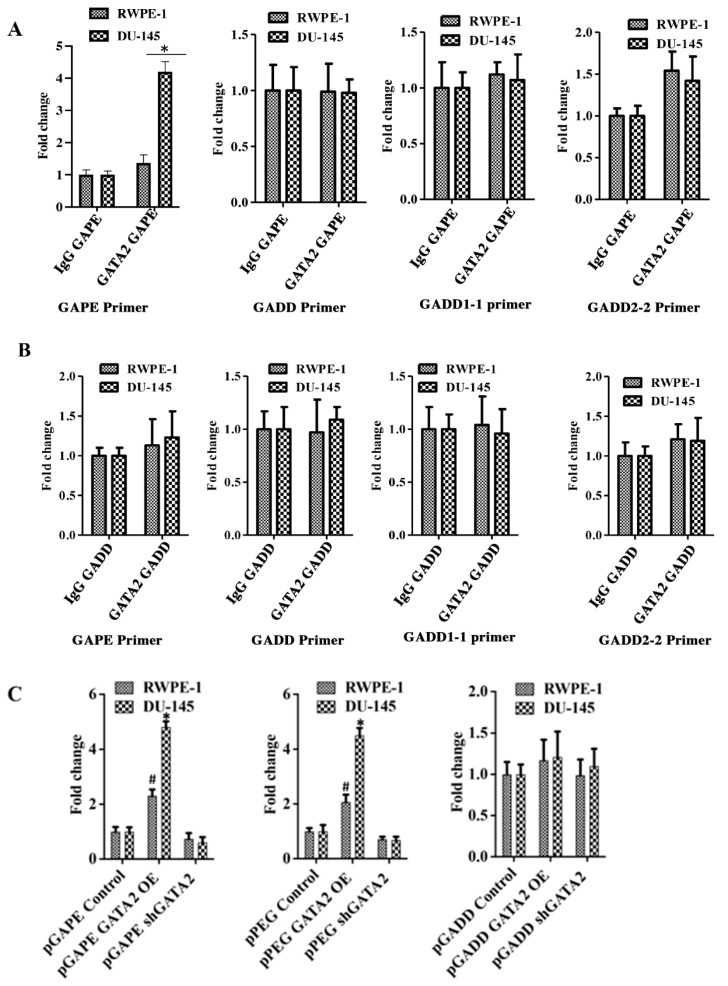
Quantitative ChIP analysis confirms cancer specificity of pGAPE. (**A**) DU-145/RWPE-1 cells were transfected with pGAPE for 48 h and used for ChIP assay. ChIP assays were performed using the GATA2 antibody and different primer sets (pGAPE, pGADD, pGADD1-1 and pGADD2-2-Prom) with the pPEG as the target for PCR amplification. (**B**) DU-145/RWPE-1 cells were transfected with pGADD for 48 h and used for ChIP assay. ChIP assays were performed using the GATA2 antibody with different primer sets (pGAPE, pGADD, pGADD1-1 and pGADD2-2) and pPEG as the target for PCR amplification. Quantitative real-time PCR (qPCR) was performed to quantify the DNA in the samples with different sets of primers (pGAPE, pGADD34, pGADD1-1 and pGADD2-2 primers) as indicated. The results presented are from three independent experiments. *, *p* < 0.01 vs. pGAPE-control. (**C**) GATA2 was either overexpressed or downregulated in DU-145/RWPE-1 cells and transfected with the indicated plasmids. ChIP assays were performed using GATA2 antibody with pGAPE primer sets using pGAPE as the target for PCR amplification (left and center panel), with pGADD primer sets with pGAPE as the target for PCR amplification (right panel). Quantitative real-time PCR (qPCR) was performed to quantify the DNA in the samples. The results presented are from three independent experiments. *, *p* < 0.01 vs. control; #, *p* < 0.05 vs. control (FDR corrected).

Chromatin immunoprecipitation (ChIP) assays confirmed an interaction between GATA2 and pGAPE in DU-145 and RWPE-1 cells (Figure 5A). DU-145 or RWPE-1 cells were transfected with either pGAPE or pGADD for 48 h, DNA was isolated after crosslinking, and chromatin fragmentation was performed using sonication. The fragmented chromatin were immunoprecipitated (pull down assay) with either nonspecific IgG or GATA2 antibody, and promoter binding to bound samples was monitored by PCR using pGAPE-, pGADD-, pGADD1-1-, or pGADD2-2-specific primers. Increased binding of GATA2 to pGAPE in DU-145 cells as compared to RWPE-1 cells was evident in comparison with pGADD or pGADD1-1 (Figure 5A,B). When pulled down with GATA2 and probed with pGAPE primers, pGAPE binding increased ~4-fold in DU-145 and ~1.5-fold in RWPE-1, whereas this binding was not altered with pGADD or pGADD1-1. pGADD2-2 primers showed a ~1.8-fold increased binding in both DU-145 and RWPE-1 (Figure 5A). GATA2 binding to the pGADD in DU-145 and RWPE-1 was similar when different primers were used (pGAPE, pGADD, pGADD1-1 or pGADD2-2) (Figure 5B). In total, these results confirm that GATA2 binding to pGAPE is higher in cancer cells.

We next determined whether changing GATA2 expression could modulate promoter binding to GATA2. RWPE-1 and DU-145 were transfected with GATA2 OE plasmid or shGATA-2 (inhibitory) plasmid for 48 h and then processed for ChIP assays. We predicted and confirmed that, following overexpression of GATA2, the binding to pGAPE/pPEG increased as compared to non-transfected cells, and conversely, GATA2 inhibition reduced binding to the pGAPE/pPEG. However, binding to the pGADD was not altered in either of these cell lines under these conditions (Supplementary Figure S5, Figure 5C). Collectively, these results advocate that pGAPE/pPEG binding to GATA2 is higher in cancer cells compared to normal cells, and when GATA2 expression is changed in these cells, binding is also altered. This demonstrates that GATA2 is an important transcription factor, which regulates pGAPE activity selectively in cancer cells. This selectivity may enhance when expressed in combination with AP1 and PEA3, which are present in pPEG [11,15,16].

### 2.7. Mutations in the FL GADD34-Prom (pT-GADD) Do Not Promote Tumor Specificity

**Figure 6:**
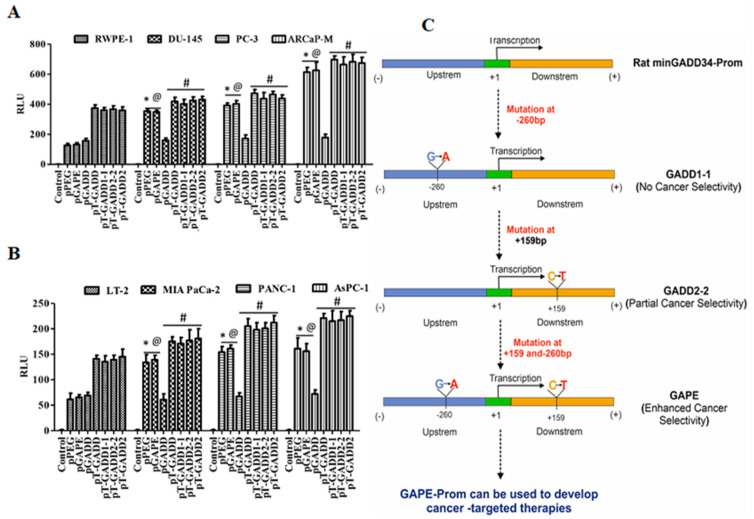
Promoter analysis of full length GADD34-Prom (pT-GADD) with single or double mutations in different cancer cells. (**A**) Immortalized human prostate epithelial (RWPE-1) and prostate cancer (DU-145, PC-3 and ARCaP-M) cells. *, *p* < 0.01 vs. RWPE-pGADD (FDR corrected); @, *p* < 0.01 vs. RWPE-pGADD (FDR corrected); #, not significant compared to PEG/GAPE between the cell lines. (**B**) Human immortalized pancreatic mesenchymal (LT-2) and pancreatic cancer (MIA PaCa-2, AsPC-1 and PANC-1) cells were transfected with PGL4-Luc (Control), pGADD34, pPEG, pGAPE, pT-GADD, pT-GADD1-1, pT-GADD2-2, or pT-GADD-2. Expression was normalized using pRL-TK, and the luminescence readings were plotted as relative luminescence units (RLU). The results presented are from three independent experiments. *, *p* < 0.01 vs. RWPE-pGADD (FDR corrected); @, *p* < 0.01 vs. RWPE-pGADD (FDR corrected); #, not significant compared to PEG/GAPE between the cell lines. (**C**) Flow chart showing the effect of different mutations in the pGADD and resultant properties. pGADD1-1 single mutation (bp −260, G-A), pGADD2-2 single mutation (bp +159, C-T) and pGAPE double mutants (bp −260 and bp +159). pGADD2-2 shows partial cancer selectivity, while pGAPE shows enhanced cancer selectivity. pGAPE can be used to generate transgene-expressing constructs that express uniquely at elevated levels in cancer cells, with minimal expression in normal cells.

To determine if a single mutation or both mutations (bp −260 or bp +159) in the full-length GADD34-Prom (pT-GADD, 1558 bp) could confer cancer selectivity, we developed total pT-GADD constructs with a single mutation at G-A, pT-GADD1-1-Luc or C-T, pT-GADD2-2-Luc, and with both the mutations, pT-GADD2-Luc (sequence information, Supplementary Data). RWPE-1 and prostate cancer (DU-145, PC-3 and ARCaP-M) cells and LT-2 and pancreatic cancer (MIA PaCa-2, AsPC-1 and PANC-1) cells were transfected with pGADD, pPEG, pGAPE, pT-GADD, pT-GADD1-1, pT-GADD2-2 and pT-GADD-2 along with PRL-TK for 48 h. As shown in Figure 6A, the relative activity of pPEG and pGAPE was ~3–4-fold higher in prostate cancer cells, in comparison with RWPE-1, whereas pT-GADD, pT-GADD1-1, pT-GADD2-2 and pT-GADD2 activity was similar to RWPE-1 with the exception of ARCaP-M cells, which were ~1.6-fold higher. Similarly, the activity of pGAPE and pPEG was ~3–4-fold higher in the pancreatic cancer cell lines in comparison to LT-2, whereas pT-GADD, pT-GADD1-1, pT-GADD2-2 and pT-GADD2 activity was similar to LT-2 (Figure 6B). In all the cell lines, pT-GADD, pT-GADD1-1, pT-GADD2-2 and pT-GADD2 activity was not significantly changed between cancer and respective normal immortal cells. pT-PEG activity was lower in normal cells as compared to cancer cells (RWPE-1 vs. DU-145, PC-3, ARCaP-M and LT2 vs. MIA PaCa-2, PANC-1 and AsPC-1), supporting its cancer specificity; however, pT-GADD did not show any cancer specificity (Supplementary Figure S6). These results suggest that the activity of pGAPE, but not the pT-GADD2 (with two mutations), is higher in cancer cells as compared to normal cells. Although total promoter activity of the pT-GADD, which reflects promoter complexity, was higher in cancer cells compared to the pGADD, no cancer selectivity was evident. Similarly, mutations in the pT-GADD did not alter its cancer selectivity. Collectively, our studies indicate that the full-length or min-GADD34-Prom does not show cancer selectivity, and both mutations are required for achieving full cancer specificity in pGAPE, but these two mutations do not promote cancer selectivity when engineered into the pT-GADD (Figure 6C).

## 3. Discussion

Cancer-selective promoters are important components of molecular medicine that can be used for disease targeting and imaging [22,27,29,30]. Both full-length and minimal pPEG exhibit cancer-selective expression in rodent and human tumors and in transformed cells, with negligible expression in normal cells [5,6,22,29,30,31,32]. Comparison of a minimal functional region of rat PEG-Prom, pPEG, to an analogous minimal region of rat GADD34-Prom, pGADD, revealed surprising similarity with only a two base pair difference. This unexpected discovery has now been scrutinized experimentally to determine the significance of these changes in mediating cancer-selective transgene expression. Mutagenic conversion of the two base pairs that differ between pGADD and pPEG results in transformation of the pGADD into the pPEG, termed the GADD34 to PEG-3 promoter (pGAPE). Comparison of the activity of the newly engineered pGAPE mutant construct with the pPEG and pGADD constructs in a series of cancer and normal cell lines confirmed that changing both of these two-point mutations in the non-specific pGADD was required for conversion to maximum enhanced cancer specificity (pGAPE). In contrast, genetically engineering a single-point mutation at base pair −260 (G-A, pGADD1-1) in pGADD failed to elicit cancer selectivity, whereas mutating the single point mutation at base pair +159 (C-T, pGADD2-2) resulted in cancer selectivity that was less than obtained after changing both point mutations. Complexity of the rat full-length GADD-34-Prom (pT-GADD) was further documented, since engineering both mutational differences found in the pGAPE in the pT-GADD did not result in cancer selectivity.

Alterations in promoter sequences can significantly alter promoter activity [33,34,35,36,37,38,39,40,41,42,43]. The most common noncoding alterations in cancer are somatic mutations in the telomerase reverse transcriptase (TERT) promoter region [33,34,35,36]. These mutations are believed to activate telomerase, consequently promoting proliferative immortality. The mutant TERT promoter is transcriptionally active and contains the H3K4me2/3 active chromatin mark and recruits the GABPA/B1 transcription factor, whereas in several cancer cell lines, the wild-type allele retains the H3K27me3 epigenetic silencing mark and is transcriptionally inactive [33]. The survivin gene contains a small number of cancer-related mutations. The survivin promoter region (−510 to +40 bp) in several malignant cancer cell lines contains a frequent C to G mutation at the −31 bp site that is absent in normal cells. This mutation is located within a cycle-dependent element (CDE) motif responsible for altering DNA-protein interactions in the CDE DNA region, which is associated with increased survivin promoter activity and endogenous survivin expression in cancer cells [38,39,40]. Moreover, a single nucleotide somatic mutation in the proximal promoter of the human TERT gene can create novel consensus sequences for transcription factors that increase TERT expression [41]. These observations place current studies in perspective and reveal how minimal changes in the sequence of specific promoters can mediate substantial changes in promoter expression, activity and transcription factor binding [33,34,35,36,37,38,39,40,41,42,43].

pPEG is principally regulated by two transcription factors, PEA-3 and AP-1 [9,15,16]. PEA-3 and AP-1 regulate the expression of a diverse array of genes involved in invasion, transformation, and tumor progression [16,44,45,46]. To identify additional transcription factors that might impact the cancer-selective activity of pGAPE, we probed small sequences from pGADD with and without mutations using the TFBIND software [28] that predicts corresponding transcription factors. The TFBIND software identified GATA2 as a transcription factor potentially associated with cancer specificity when simultaneously present with both the mutations in pGAPE. GATA2, a member of the zinc finger transcription factor family, has been identified as a critical regulator of hematopoietic stem cell growth, differentiation, and survival [47,48,49,50]. GATA2 expression is linked to hematologic pathologies as well as the proliferation and progression of solid tumors. Overexpression of GATA2 correlates with development of breast cancer by negatively regulating the transcription of phosphatase and tensin homologue (PTEN) [51]. In prostate cancer, increased GATA2 expression associates with tumor progression, and GATA2 has been proposed as a prominent factor in the regulation of androgen receptor-related genes. Apart from hematopoietic systems, GATA2 expression is found in the brain, kidney, endothelial cells, placenta, pituitary gland, prostate, adipocytes, and lungs [52,53,54]. GATA2 binds and regulates activity of the promoters of several endothelial-specific genes, including Platelet and Endothelial Cell Adhesion Molecule1 (PECAM1) [55] and Endothelin 1 (EDN1) [56], as well as vascular endothelial growth factor receptor 2 (VEGFR2) expression during vascular development and angiogenesis [57,58]. GATA2 expression was enhanced in all of the cancer cell lines tested in comparison with their normal counterparts. Additionally, we found that GATA2 overexpression enhanced GAPE cancer specificity, and conversely, GATA2 inhibition reduced GAPE cancer specificity. ChIP analysis confirmed that GATA2 binds to pGAPE, which is enhanced when GATA2 is overexpressed and diminished upon GATA2 knockdown. Using different primer sets in our ChIP assay, we further showed that GATA2 marginally binds to pGADD and pGADD1-1 and partially binds to pGADD2-2, whereas a significant enhanced binding was evident in pGAPE.

As observed with the pPEG [22], pGAPE could non-invasively deliver an imaging agent to cancers in several preclinical immune-compromised and immune-competent prostate and breast cancer mouse models. Additionally, we were able to image metastatic lesions in experimental metastatic models of breast and prostate cancer. Similar to other cancer-selective imaging agents, pGAPE can be used for preoperative planning, intraoperative management, and treatment monitoring. The pGAPE imaging system can also be used as a theranostic agent by using an internal ribosome entry site or another technique that allows for tandem gene expression [32]. Because the human genome lacks a pPEG homolog, including the human pGADD homolog’s promoter/enhancer region, pGAPE usage in humans is anticipated to produce minimal background signals. These findings suggest that pGAPE could provide a practical methodology for imaging and possibly image-guided therapy of various cancers.

Tissue- and cancer-selective/specific promoters also provide functional tools to potentially regulate virus replication in a conditional manner, including adenovirus, herpesvirus, human immunodeficiency virus, simian immunodeficiency virus, and others [29,30,31,32,59,60]. A human telomerase promoter and an insulin promoter, which are controlled by PDX-1, have been used to deliver transgenes to pancreatic cancer cells [61,62]. A more global anti-cancer approach involves conditionally replicating adenoviruses (Ads), both Ad.5 and Ad.5/3 chimeric viruses, and engineering them to use the pPEG (or pGAPE) to control Ad E1A and E1B expression. These viruses have also been engineered to conditionally express a tumor suppressor gene, *mda-7/IL-24* [63], under control of a CMV promoter, referred to as a Cancer Terminator virus (*CTV*). The *CTV* induces profound anticancer activity in vitro and in vivo in diverse cancer indications [29,30,31,32]. Considering the broad-spectrum cancer specificity of pPEG and pGAPE, these promoters represent appropriate tools to develop next generation conditionally replication-competent Ads (CRADs) that replicate preferentially in both rodent and human cancer cells. This promoter can also be used to develop a next generation “theranostic” cancer terminator virus that retains cancer-selective replication, targeted therapeutic cytokine expression, and non-invasive imaging potential [32].

In summary, we document that two-point mutations are critical to convert a non-cancer-specific pGADD into a cancer-selective pGAPE. A single mutational change at bp +159 of pGADD to that of the pPEG also promotes cancer selectivity both in vitro and in vivo. Additionally, we define a new transcription factor, GATA2, to which binding to the GAPE-Prom is indispensable for cancer-selective expression and activity. This newly engineered pGAPE embodies all the necessary features that make it an ideal tool to regulate transgene expression in a cancer-selective manner to both deliver imaging agents noninvasively to follow tumor promotion and metastasis and create CRADs expressing replicative functions uniquely in cancers.

## 4. Materials and Methods

### 4.1. Cell Lines and Reagents

Immortalized human prostate epithelial (RWPE-1) and prostate cancer (DU-145, PC-3 and ARCaP-M) cells, human immortalized pancreatic mesenchymal (LT-2) and pancreatic cancer (MIA PaCa-2, AsPC-1 and PANC-1) cells, early passage primary human mammary epithelial (HMEC) and breast cancer (MDA-MB-231 and SUM159) cells, H-TERT immortalized primary human fetal astrocytes (IM-PHFA) and neuroblastoma (SK-N-AS, NB-1691 and SK-N-SH) cells were cultured as described previously [16,31,32,64,65]. Suggested culture media supplemented with 10% FBS, 50 units/mL penicillin, and 50 μg/mL streptomycin (Life Technologies Inc., Carlsbad, CA, USA) were used for cell cultures. Cells were incubated in a humidified incubator with 5% CO_2_ at 37 °C. Cells were validated for authenticity in prior studies [16,31,32,64,65] and were routinely evaluated for mycoplasma contamination [16,31,32,64].

Antibodies specific for: GATA2, Control Rabbit IgG (Cell signaling technology, Danvers, MA, USA), horseradish peroxidase (HRP)-conjugated secondary antibodies (Dako, Santa Clara, CA, USA), and β-actin (#NB600-501; Novus Biologicals, Inc., Littleton, CO, USA) were used in this study. SimpleChIP^®^ Plus Enzymatic Chromatin IP Kit (Magnetic Beads) was purchased from Cell signaling technology. Dual Luciferase Reporter Assay Kit was obtained from Promega (Madison, WI, USA) and used in the study. Lipofectamine 2000 (Invitrogen, Waltham, MA) and Invivo JetPEI (Polyplus, Genesee Scientific Corporation, San Diego, CA, USA) and transfection reagents, D-Luciferin Potassium Salt Bioluminescent Substrate (Waltham, MA, USA), Agilent Quickchange II site directed mutagenesis kit (Santa Clara, CA, USA) were used in the study.

### 4.2. Plasmid Promoter Construction and Mutagenesis

Rat progression-elevated gene-3 (PEG-3) was isolated from H5ts125-transformed rat embryo cancer cells by subtraction hybridization and is not expressed in normal rodent or human cells [5,16]. The minimal active promoter region of the PEG-3 gene (pPEG) was isolated, and expression was shown to be cancer-selective [9,15,16]. The PEG-3 gene, present only in transformed rodent cells, was found to be similar to the naturally occurring rat GADD34 gene [5]. Two base pairs that differed between pPEG and minGADD34 (pGADD) were modified converting pGADD to pPEG, called pGAPE (minGADD34 to minPEG-3 promoter), using an Agilent Quickchange II site directed mutagenesis kit following the manufacturer’s protocol. Using standard PCR primers (list of primers below) we performed polymerase chain reactions (PCR) to amplify the promoter with the specific mutated base pairs. The PCR product was cloned into pGL4.14 using restriction enzymes BglII and KpnI. The respective pPEG/pGADD/pGAPE mutant constructs were transfected into cells along with the plasmid pRL-TK (Renilla, as a control) using Lipofectamine 2000 according to the manufacturer’s instructions. Cells were lysed, and luminescence was measured using the Dual-Glo luciferase assay system [9,16]. Mutating these 2 base pairs on the converted GADD34 promoter back to the base pairs present in the PEG-3 promoter restored cancer-selective activity.

### 4.3. Primers for Mutating the First Base Pair of the GADD34 Promoter

pGADD34-F

CGGGGTACCGAAAGAGAAAGAGAATGGGACAGCA

pGADD34-R

CGGAAGATCT GGTCCGGTTCGGTTTGCCAAAAGCGGTC

### 4.4. Primers for Mutating the Second Base Pair of the GADD34 Promoter

pGADD-F

CGGGGTACCGAAAGAGAAAGAGAATGGGACGGCA

pGADD-R

CGGAAGATCTGGTCCGGTTCGGTTTGCCAAAAGCGATC

### 4.5. Sequences of the Minimal Promoters Described in the Study

#### 4.5.1. Rat PEG-3 Minimal Promoter Sequence

GAAAGAGAAAGAGAATGGGACAGCATGTGACTGCCTGATGAAGTTGGCGTGCTTGCTCAAAAGTTCTGCGAGATTGACGGCTCTCTGGATTTGAGCCAAGGACACGCCTGGGAAGCCACGGTGACCTCACAAGGCCCGGAATCTCCGCGAGAATTTCAGTGTTGTTTTCCTCTCTCCACCTTTCTCAGGGACTTCCGAAACTCCGCCTCTCCGGTGACGTCAGCATAGCGCTGCGTCAGACTATAAACTCCCGGGTGATCGTGTTGGCGCAGATTGACTCAGTTCGCAGCTTGTGGAAGATTACATGCGAGACCCCGCGCGACTCCGCATCCCTTTGCCGGGACAGCCTTTGCGACAGCCCGTGAGACATCACGTCCCCGAGCCCCACGCCTGAGGGCGACATGAACGCGCTGGCCTTGAGAGCAATCCGGACCCACGATCGCTTTTGGCAAACCGAACCGGACC

#### 4.5.2. Rat GADD34 Minimal Promoter Sequence

GAAAGAGAAAGAGAATGGGACGGCATGTGACTGCCTGATGAAGTTGGCGTGCTTGCTCAAAAGTTCTGCGAGATTGACGGCTCTCTGGATTTGAGCCAAGGACACGCCTGGGAAGCCACGGTGACCTCACAAGGCCCGGAATCTCCGCGAGAATTTCAGTGTTGTTTTCCTCTCTCCACCTTTCTCAGGGACTTCCGAAACTCCGCCTCTCCGGTGACGTCAGCATAGCGCTGCGTCAGACTATAAACTCCCGGGTGATCGTGTTGGCGCAGATTGACTCAGTTCGCAGCTTGTGGAAGATTACATGCGAGACCCCGCGCGACTCCGCATCCCTTTGCCGGGACAGCCTTTGCGACAGCCCGTGAGACATCACGTCCCCGAGCCCCACGCCTGAGGGCGACATGAACGCGCTGGCCTTGAGAGCAATCCGGACCCACGACCGCTTTTGGCAAACCGAACCGGACC

#### 4.5.3. Mutated Rat GADD34 Minimal Promoter Sequence (Both Base Pairs Mutated)

GAAAGAGAAAGAGAATGGGACAGCATGTGACTGCCTGATGAAGTTGGCGTGCTTGCTCAAAAGTTCTGCGAGATTGACGGCTCTCTGGATTTGAGCCAAGGACACGCCTGGGAAGCCACGGTGACCTCACAAGGCCCGGAATCTCCGCGAGAATTTCAGTGTTGTTTTCCTCTCTCCACCTTTCTCAGGGACTTCCGAAACTCCGCCTCTCCGGTGACGTCAGCATAGCGCTGCGTCAGACTATAAACTCCCGGGTGATCGTGTTGGCGCAGATTGACTCAGTTCGCAGCTTGTGGAAGATTACATGCGAGACCCCGCGCGACTCCGCATCCCTTTGCCGGGACAGCCTTTGCGACAGCCCGTGAGACATCACGTCCCCGAGCCCCACGCCTGAGGGCGACATGAACGCGCTGGCCTTGAGAGCAATCCGGACCCACGATCGCTTTTGGCAAACCGAACCGGACC

#### 4.5.4. Mutated Rat GADD34 Minimal Promoter Sequence (1st Base Pair Mutated)

GAAAGAGAAAGAGAATGGGACAGCATGTGACTGCCTGATGAAGTTGGCGTGCTTGCTCAAAAGTTCTGCGAGATTGACGGCTCTCTGGATTTGAGCCAAGGACACGCCTGGGAAGCCACGGTGACCTCACAAGGCCCGGAATCTCCGCGAGAATTTCAGTGTTGTTTTCCTCTCTCCACCTTTCTCAGGGACTTCCGAAACTCCGCCTCTCCGGTGACGTCAGCATAGCGCTGCGTCAGACTATAAACTCCCGGGTGATCGTGTTGGCGCAGATTGACTCAGTTCGCAGCTTGTGGAAGATTACATGCGAGACCCCGCGCGACTCCGCATCCCTTTGCCGGGACAGCCTTTGCGACAGCCCGTGAGACATCACGTCCCCGAGCCCCACGCCTGAGGGCGACATGAACGCGCTGGCCTTGAGAGCAATCCGGACCCACGACCGCTTTTGGCAAACCGAACCGGACC

#### 4.5.5. Mutated Rat GADD34 Minimal Promoter Sequence (2nd Base Pair Mutated)

GAAAGAGAAAGAGAATGGGACGGCATGTGACTGCCTGATGAAGTTGGCGTGCTTGCTCAAAAGTTCTGCGAGATTGACGGCTCTCTGGATTTGAGCCAAGGACACGCCTGGGAAGCCACGGTGACCTCACAAGGCCCGGAATCTCCGCGAGAATTTCAGTGTTGTTTTCCTCTCTCCACCTTTCTCAGGGACTTCCGAAACTCCGCCTCTCCGGTGACGTCAGCATAGCGCTGCGTCAGACTATAAACTCCCGGGTGATCGTGTTGGCGCAGATTGACTCAGTTCGCAGCTTGTGGAAGATTACATGCGAGACCCCGCGCGACTCCGCATCCCTTTGCCGGGACAGCCTTTGCGACAGCCCGTGAGACATCACGTCCCCGAGCCCCACGCCTGAGGGCGACATGAACGCGCTGGCCTTGAGAGCAATCCGGACCCACGATCGCTTTTGGCAAACCGAACCGGACC

Sequences of the full-length GADD promoters with mutations are described in the supplementary information.

### 4.6. Promoter Assay

The prostate cancer, pancreatic cancer, breast cancer, and neuroblastoma cell lines and primary or immortalized normal cells were plated in 24-well plates (BD Biosciences) and transfected using Lipofectamine 2000 (Invitrogen) according to the manufacturer’s protocol. The indicated cells were transfected with Luc reporter constructs pPEG-Luc, pGADD-Luc, pGAPE-Luc or a empty vector (control). Luminescence was normalized for transfection efficiency by co-transfection with a vector expressing Renilla luciferase (Luc). After 48 h of transfection, the expression level of the Luc reporter was measured by the Dual Luciferase Reporter Assay Kit (Promega) [9,16].

### 4.7. Western Blotting

Western blotting analysis was performed as described previously [5,11,64]. RIPA (radio-immunoprecipitation assay) lysis buffer containing phosphatase and protease inhibitors was used to lyse the parental or GATA2 OE or shGATA2 cells. Total protein was measured using BCA reagent, and equal total protein was resolved by SDS-PAGE and transferred to a polyvinylidene difluoride (PVDF) membrane. The membrane was blocked with 5% non-fat dry milk or 5% BSA for 1 h. Blocked membranes were incubated overnight with primary antibodies followed by HRP-conjugated secondary antibodies. An ECL reagent was used to detect chemiluminescent signals and captured using X-Ray films. Equal loading was confirmed by reprobing all the blots with β-actin antibody. The original western blots see Figure S7.

### 4.8. Systemic Delivery of Plasmid Constructs

Low-molecular-weight l-PEI–based cationic polymer, in vivo jetPEI (Polyplus Transfection), was used for gene delivery [15,27]. The DNA-PEI polyplex was formed according to the manufacturer’s instructions. For systemic delivery, 40 μg of DNA and 4.8 μL of 150 mmol/L in vivo jetPEI were separately diluted in endotoxin free 5% (*wt/vol*) glucose. The glucose solutions of DNA and l-PEI polymer were then mixed together to give an N:P ratio (the number of nitrogen residues of in vivo jetPEI per number of phosphate groups of DNA) of 6:1 in a total volume of 400 μL. The DNA-PEI polyplex was injected intravenously as two 200 μL injections with a 5 min interval.

### 4.9. Bioluminescence Imaging

In vivo BLI was conducted at 24 and 48 h after the systemic delivery of reporter genes [22,27]. All the mice in the study were imaged with the IVIS Spectrum [32,64]. For each imaging session, mice were injected intraperitoneally with 150 mg/kg D-luciferin, potassium salt under anesthesia using a 2.0% isoflurane/oxygen mixture. Ex vivo BLI was conducted within 10 min of necropsy. Living Image 2.5 and Living Image 3.1 software were used for image acquisition and analysis.

### 4.10. Chromatin Immunoprecipitation

ChIP assays were performed as described previously [66]. Briefly, cells were fixed in 1% paraformaldehyde for 15 min, and chromatin was sheared to an average size of 300 bp by sonication. Lysates were incubated with 0.5 μg anti-GATA2 (Cell signaling technologies) or control rabbit IgG bound to Protein G Dyna beads (Cell Signaling technologies) overnight, subsequently washed with low- and high-salt buffers and eluted with 0.1 mol/L NaHCO_3_ in 1% SDS. Primer pairs are listed below.

### 4.11. Primers for ChIP Analysis

The following primers were used in the ChIP assay:

GADD F- GAA AGA GAA AGA GAA TGG GAC G

GADD R- GTC CGG TTC GGT TTG CCA AAA GCG G

GAPE F- GAA AGA GAA AGA GAA TGG GAC A

GAPE R- GTC CGG TTC GGT TTG CCA AAA GCG A

GAD1-1 F- GAA AGA GAA AGA GAA TGG GAC A

GADD1-1 R- GTC CGG TTC GGT TTG CCA AAA GCG G

GADD2-2 F- GAA AGA GAA AGA GAA TGG GAC G

GADD2-2 R- GTC CGG TTC GGT TTG CCA AAA GCG A

### 4.12. Statistical Methods

Statistical analysis was performed using SPSS 22.0 software (SPSS, Chicago, IL, USA), and statistical graphs were generated using GraphPad Prism (GraphPad Software Inc., San Diego, CA, USA). The generalized odds ratios (ORs) and 95% confidence interval (CI) of the promoters were calculated. The multiple comparisons were performed using false discovery rate (FDR) correction. False discovery rate (FDR) correction was used to analyze the promoter activity among the different promoters.

## Data Availability

The data presented in this study are available on request from the corresponding author.

## Supplementary Materials

The following supporting information can be downloaded at: https://www.mdpi.com/article/10.3390/cancers14061497/s1. Figure S1: Sequence comparisons between rat pPEG (minPEG-Prom) and rat pGADD (min-GADD34-Prom), Figure S2: Tumor imaging with pGADD1-1-PEI or pGADD2-2-PEI, Figure S3: Transcription factors involved in cancer specificity, Figure S4: Densitometry analysis of Western blots from Figure 4, Figure S5: ChIP assays using GATA2 antibody and different primer sets, Figure S6: Activity of full-length GADD and PEG promoter in normal and cancer cells. Figure S7, Original western blots for Figure 4.

## References

[1] Vogelstein B., Kinzler K.W. (1993). The multistep nature of cancer. Trends Genet..

[2] Carbone M., Pass H.I. (2004). Multistep and multifactorial carcinogenesis: When does a contributing factor become a carcinogen?. Semin. Cancer Biol..

[3] Chaffer C.L., Weinberg R.A. (2015). How does multistep tumorigenesis really proceed?. Cancer Discov..

[4] Adolph K.W., Reddy P.G., Su Z.-Z., Fisher P.B., Adolph K.W. (1993). Chromosome and Genetic Analysis: Methods in Molecular Genetics.

[5] Su Z.Z., Shi Y., Fisher P.B. (1997). Subtraction hybridization identifies a transformation progression-associated gene PEG-3 with sequence homology to a growth arrest and DNA damage-inducible gene. Proc. Natl. Acad. Sci. USA.

[6] Su Z.-Z., Goldstein N.I., Jiang H., Wang M.-N., Duigou G.J., Young C.S.H., Fisher P.B. (1999). PEG-3, a nontransforming cancer progression gene, is a positive regulator of cancer aggressiveness and angiogenesis. Proc. Natl. Acad. Sci. USA.

[7] Su Z.-Z., Emdad L., Sarkar D., Randolph A., Valerie K., Yacoub A., Dent P., Fisher P.B. (2005). Potential molecular mechanism for rodent tumorigenesis: Mutational generation of Progression Elevated Gene-3 (PEG-3). Oncogene.

[8] Otsuka R., Harada N., Aoki S., Shirai K., Nishitsuji K., Nozaki A., Hatakeyama A., Shono M., Mizusawa N., Yoshimoto K. (2015). C-terminal region of GADD34 regulates eIF2α dephosphorylation and cell proliferation in CHO-K1 cells. Cell Stress Chaperon..

[9] Su Z., Shi Y., Fisher P.B. (2000). Cooperation between AP1 and PEA3 sites within the progression elevated gene-3 (PEG-3) promoter regulate basal and differential expression of PEG-3 during progression of the oncogenic phenotype in transformed rat embryo cells. Oncogene.

[10] Hollander M.C., Zhan Q., Bae I., Fornace A.J. (1997). Mammalian GADD34, an apoptosis- and DNA damage-inducible gene. J. Biol. Chem..

[11] Sarkar D., Su Z.-Z., Lebedeva I.V., Sauane M., Gopalkrishnan R.V., Valerie K., Dent P., Fisher P.B. (2002). *mda*-7 (IL-24) mediates selective apoptosis in human melanoma cells by inducing the coordinated overexpression of the GADD family of genes by means of p38 MAPK. Proc. Natl. Acad. Sci. USA.

[12] Zhan Q., Lord K.A., Alamo I., Hollander M.C., Carrier F., Ron D., Kohn K.W., Hoffman B., Liebermann D.A., Fornace A.J. (1994). The gadd and MyD genes define a novel set of mammalian genes encoding acidic proteins that synergistically suppress cell growth. Mol. Cell. Biol..

[13] Hollander M.C., Poola-Kella S., Fornace A.J. (2003). Gadd34 functional domains involved in growth suppression and apoptosis. Oncogene.

[14] Yagi A., Hasegawa Y., Xiao H., Haneda M., Kojima E., Nishikimi A., Hasegawa T., Shimokata K., Isobe K. (2003). GADD34 induces p53 phosphorylation and p21/WAF1 transcription. J. Cell. Biochem..

[15] Su Z., Shi Y., Friedman R., Qiao L., McKinstry R., Hinman D., Hasegawa T., Shimokata K., Isobe K. (2001). PEA3 sites within the progression elevated gene-3 (PEG-3) promoter and mitogen-activated protein kinase contribute to differential PEG-3 expression in Ha-ras and v-raf oncogene transformed rat embryo cells. Nucleic Acids Res..

[16] Su Z.-Z., Sarkar D., Emdad L., Duigou G.J., Young C.S.H., Ware J., Randolph A., Valerie K., Fisher P.B. (2005). Targeting gene expression selectively in cancer cells by using the progression-elevated gene-3 promoter. Proc. Natl. Acad. Sci. USA.

[17] Xu Y., Krishnan A., Wan X.S., Majima H., Yeh C.-C., Ludewig G., Kasarskis E.J., St.Clair D.K. (1999). Mutations in the promoter reveal a cause for the reduced expression of the human manganese superoxide dismutase gene in cancer cells. Oncogene.

[18] Bell R.J.A., Rube H.T., Xavier-Magalhães A., Costa B., Mancini A., Song J., Costello J.F. (2016). Understanding TERT Promoter Mutations: A Common Path to Immortality. Mol. Cancer Res..

[19] Lee D.D., Komosa M., Sudhaman S., Leão R., Zhang C.H., Apolonio J.D., Hermanns T., Wild P.J., Klocker H., Nassiri F. (2021). Dual role of allele-specific DNA hypermethylation within the TERT promoter in cancer. J. Clin. Investig..

[20] Horn S., Figl A., Rachakonda P.S., Fischer C., Sucker A., Gast A., Kadel S., Moll I., Nagore E., Hemminki K. (2013). TERT Promoter Mutations in Familial and Sporadic Melanoma. Science.

[21] Gupta S., Won H., Chadalavada K., Nanjangud G.J., Chen Y.-B., Al-Ahmadie H.A., Fine S.W., Sirintrapun S.J., Strong V.E., Raj N. (2021). TERT Copy Number Alterations, Promoter Mutations and Rearrangements in Adrenocortical Carcinomas. Endocr. Pathol..

[22] Bhang H.E., Gabrielson K.L., Laterra J., Fisher P.B., Pomper M.G. (2011). Tumor-specific imaging through progression elevated gene-3 promoter-driven gene expression. Nat. Med..

[23] Menezes M.E., Das S.K., Emdad L., Windle J., Wang X.-Y., Sarkar D., Fisher P.B. (2014). Genetically Engineered Mice as Experimental Tools to Dissect the Critical Events in Breast Cancer. Adv. Cancer Res..

[24] Sakamoto K., Schmidt J.W., Wagner K.U. (2015). Mouse models of breast cancer. Methods Mol. Biol..

[25] Valkenburg K.C., Williams B.O. (2011). Mouse models of prostate cancer. Prostate Cancer.

[26] Ellwood-Yen K., Graeber T., Wongvipat J., Iruela-Arispe M., Zhang J., Matusik R., Thomas G., Sawyers C.L. (2003). Myc-driven murine prostate cancer shares molecular features with human prostate tumors. Cancer Cell.

[27] Bhatnagar A., Wang Y., Mease R.C., Gabrielson M., Sysa P., Minn I., Green G., Simons B., Gabrielson K., Sarkar S. (2014). AEG-1 Promoter–Mediated Imaging of Prostate Cancer. Cancer Res..

[28] Shima A., Matsuoka H., Yamaoka A., Michihara A. (2021). Transcription of CLDND1 in human brain endothelial cells is regulated by the myeloid zinc finger 1. Clin. Exp. Pharmacol. Physiol..

[29] Sarkar D., Su Z.Z., Vozhilla N., Park E.S., Gupta P., Fisher P.B. (2005). Dual cancer-specific targeting strategy cures primary and distant breast carcinomas in nude mice. Proc. Natl. Acad. Sci. USA.

[30] Das S.K., Sarkar S., Dash R., Dent P., Wang X.-Y., Sarkar D., Fisher P.B. (2012). Cancer Terminator Viruses and Approaches for Enhancing Therapeutic Outcomes. Adv. Cancer Res..

[31] Sarkar D., Su Z.-Z., Vozhilla N., Park E.S., Randolph A., Valerie K., Fisher P.B. (2005). Targeted Virus Replication Plus Immunotherapy Eradicates Primary and Distant Pancreatic Tumors in Nude Mice. Cancer Res..

[32] Bhoopathi P., Pradhan A.K., Maji S., Das S.K., Emdad L., Fisher P.B. (2021). Theranostic Tripartite Cancer Terminator Virus for Cancer Therapy and Imaging. Cancers.

[33] Stern J.L., Theodorescu D., Vogelstein B., Papadopoulos N., Cech T.R. (2015). Mutation of the TERT promoter, switch to active chromatin, and monoallelic TERT expression in multiple cancers. Genes Dev..

[34] Elliott K., Larsson E. (2021). Non-coding driver mutations in human cancer. Nat. Rev. Cancer.

[35] Maturo M.G., Rachakonda S., Heidenreich B., Pellegrini C., Srinivas N., Requena C., Serra-Guillen C., Llombart B., Sanmartin O., Guillen C. (2020). Coding and noncoding somatic mutations in candidate genes in basal cell carcinoma. Sci. Rep..

[36] Colebatch A.J., Dobrovic A., Cooper W.A. (2019). TERT gene: Its function and dysregulation in cancer. J. Clin. Pathol..

[37] Zhang M., Yang J., Li F. (2006). Transcriptional and post-transcriptional controls of survivin in cancer cells: Novel approaches for cancer treatment. J. Exp. Clin. Cancer Res..

[38] Xu Y., Fang F., Ludewig G., Jones G., Jones D. (2004). A mutation found in the promoter region of the human survivin gene is correlated to overexpression of survivin in cancer cells. DNA Cell Biol..

[39] Cheng Q., Ling X., Haller A., Nakahara T., Yamanaka K., Kita A., Koutoku H., Takeuchi M., Brattain M.G., Li F. (2012). Suppression of survivin promoter activity by YM155 involves disruption of Sp1-DNA interaction in the survivin core promoter. Int. J. Biochem. Mol. Biol..

[40] Rafatmanesh A., Behjati M., Mobasseri N., Sarvizadeh M., Mazoochi T., Karimian M. (2020). The survivin molecule as a double-edged sword in cellular physiologic and pathologic conditions and its role as a potential biomarker and therapeutic target in cancer. J. Cell. Physiol..

[41] Min J., Shay J.W. (2016). TERT Promoter Mutations Enhance Telomerase Activation by Long-Range Chromatin Interactions. Cancer Discov..

[42] Shay J.W. (2016). Role of Telomeres and Telomerase in Aging and Cancer. Cancer Discov..

[43] Bell R.J., Rube H.T., Kreig A., Mancini A., Fouse S.D., Nagarajan R.P., Choi S., Hong C., He D., Pekmezci M. (2015). Cancer. The transcription factor GABP selectively binds and activates the mutant TERT promoter in cancer. Science.

[44] Cowden Dahl K.D., Zeineldin R., Hudson L.G. (2007). PEA3 is necessary for optimal epidermal growth factor receptor-stimulated matrix metalloproteinase expression and invasion of ovarian tumor cells. Mol. Cancer Res..

[45] Kherrouche Z., Monté D., Werkmeister E., Stoven L., de Launoit Y., Cortot A.B., Tulasne D., Chotteau-Lelievre A. (2015). PEA3 transcription factors are downstream effectors of Met signaling involved in migration and invasiveness of Met-addicted tumor cells. Mol. Oncol..

[46] Shen Q., Uray I.P., Li Y., Krisko T.I., Strecker T.E., Kim H.-T., Brown P.H. (2007). The AP-1 transcription factor regulates breast cancer cell growth via cyclins and E2F factors. Oncogene.

[47] Gao J., Chen Y.H., Peterson L.C. (2015). GATA family transcriptional factors: Emerging suspects in hematologic disorders. Exp. Hematol. Oncol..

[48] Menendez-Gonzalez J.B., Vukovic M., Abdelfattah A., Saleh L., Almotiri A., Thomas L.A., Agirre-Lizaso A., Azevedo A., Menezes A.C., Tornillo G. (2019). Gata2 as a Crucial Regulator of Stem Cells in Adult Hematopoiesis and Acute Myeloid Leukemia. Stem Cell Rep..

[49] Rodrigues N.P., Tipping A.J., Wang Z., Enver T. (2012). GATA-2 mediated regulation of normal hematopoietic stem/progenitor cell function, myelodysplasia and myeloid leukemia. Int. J. Biochem. Cell Biol..

[50] Fu Y.-K., Tan Y., Wu B., Dai Y.-T., Xu X.-G., Pan M.-M., Chen Z.-W., Qiao N., Wu J., Jiang L. (2021). Gata2-L359V impairs primitive and definitive hematopoiesis and blocks cell differentiation in murine chronic myelogenous leukemia model. Cell Death Dis..

[51] Wang Y., He X., Ngeow J., Eng C. (2012). GATA2 negatively regulates PTEN by preventing nuclear translocation of androgen receptor and by androgen-independent suppression of PTEN transcription in breast cancer. Hum. Mol. Genet..

[52] Yamamoto M., Takahashi S., Onodera K., Muraosa Y., Engel J.D. (1997). Upstream and downstream of erythroid transcription factor GATA-1. Genes Cells.

[53] Rodriguez-Bravo V., Carceles-Cordon M., Hoshida Y., Cordon-Cardo C., Galsky M.D., Domingo-Domenech J. (2017). The role of GATA2 in lethal prostate cancer aggressiveness. Nat. Rev. Urol..

[54] Tremblay M., Sanchez-Ferras O., Bouchard M. (2018). GATA transcription factors in development and disease. Development.

[55] Gumina R.J., Kirschbaum N.E., Piotrowski K., Newman P.J. (1997). Characterization of the human platelet/endothelial cell adhesion molecule-1 promoter: Identification of a GATA-2 binding element required for optimal transcriptional activity. Blood.

[56] Lee M.E., Temizer D.H., Clifford J.A., Quertermous T. (1991). Cloning of the GATA-binding protein that regulates endothelin-1 gene expression in endothelial cells. J. Biol. Chem..

[57] Kappel A., Schlaeger T.M., Flamme I., Orkin S.H., Risau W., Breier G. (2000). Role of SCL/Tal-1, GATA, and ets transcription factor binding sites for the regulation of flk-1 expression during murine vascular development. Blood.

[58] Coma S., Allard-Ratick M., Akino T., van Meeteren L.A., Mammoto A., Klagsbrun M. (2013). GATA2 and Lmo2 control angiogenesis and lymphangiogenesis via direct transcriptional regulation of neuropilin-2. Angiogenesis.

[59] Saukkonen K., Hemminki A. (2004). Tissue-specific promoters for cancer gene therapy. Expert Opin. Biol. Ther..

[60] Lopez M.V., Cafferata E.G., Viale D.L., Podhajcer O.L. (2017). Synthetic Tumor Specific Promoters for Transcriptional Regulation of Viral Replication. Methods Mol. Biol..

[61] Liu S., Wang X.P., Brunicardi F.C. (2007). Enhanced cytotoxicity of RIPTK gene therapy of pancreatic cancer via PDX-1 co-delivery. J. Surg. Res..

[62] Fillat C., Jose A., Bofill-Deros X., Mato-Berciano A., Maliandi M.V., Sobrevals L. (2011). Pancreatic cancer gene therapy: From molecular targets to delivery systems. Cancers.

[63] Fisher P.B. (2005). Is mda-7/IL-24 a “magic bullet” for cancer?. Cancer Res..

[64] Bhoopathi P., Pradhan A.K., Bacolod M.D., Emdad L., Sarkar D., Das S.K., Fisher P.B. (2019). Regulation of neuroblastoma migration, invasion, and in vivo metastasis by genetic and pharmacological manipulation of MDA-9/Syntenin. Oncogene.

[65] Quinn B.A., Dash R., Sarkar S., Azab B., Bhoopathi P., Das S.K., Emdad L., Wei J., Pellecchia M., Sarkar D. (2015). Pancreatic Cancer Combination Therapy Using a BH3 Mimetic and a Synthetic Tetracycline. Cancer Res..

[66] Sitcheran R., Gupta P., Fisher P.B., Baldwin A.S. (2005). Positive and negative regulation of EAAT2 by NF-kappaB: A role for N-myc in TNFalpha-controlled repression. EMBO J..

