# Supplementary material for: Conversion of a Non-Cancer-Selective Promoter into a Cancer-Selective Promoter
Source: Cancers (Basel). 2022 Mar 15;14(6):1497. doi: 10.3390/cancers14061497 (PMC8946048; doi:10.3390/cancers14061497)
Supplement: Supplementary file 1 [file cancers-14-01497-s001.zip › cancers-1569542-supplementary.pdf]

Supplementary Material: Conversion of a Non-Cancer-Selective Promoter into a Cancer-Selective Promoter

Praveen Bhoopathi, Anjan K. Pradhan, Amit Kumar, Santanu Maji, Padmanabhan Mannangatti, Xiaoyan Deng, Dipankar Bandyopadhyay, Devanand Sarkar, Xiang-Yang Wang, Joseph W. Landry, Swadesh K. Das, Luni Emdad and Paul B. Fisher

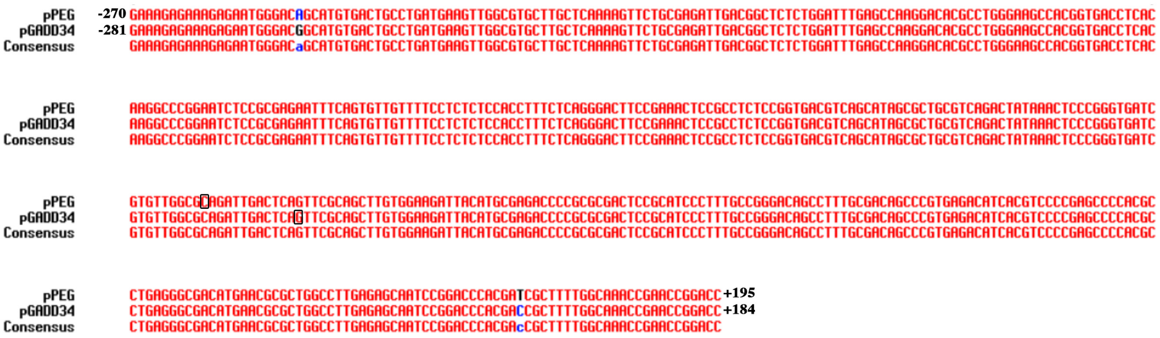

Figure S1. Sequence comparisons between rat pPEG (minPEG-Prom) and rat pGADD (minGADD34-Prom). The base changes in the promoter are marked in blue and site-directed mutagenesis was performed to mutate the specified bases in the pGADD to convert it into the pGAPE. At base pair -260, G was converted to A in the pGADD and in base pair +159, C was converted to T in pGADD. The pGADD with both the mutations, rendering it identical to pPEG, called pGAPE. Black box: Gene Start site (+1).

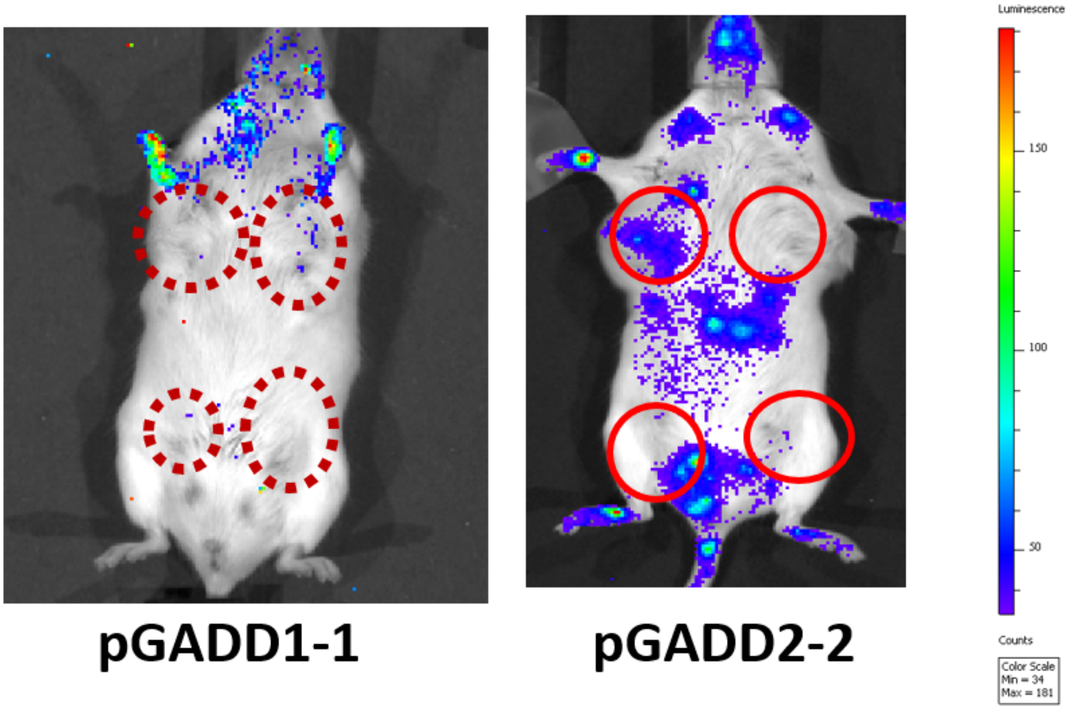

Figure S2. Tumor bearing transgenic PyMT mice were intravenously injected with pGADD1-1-PEI or pGADD2-2-PEI and bioluminescent imaging (BLI) was performed 48 h later by IVIS. Only a subset of tumors identified as hatched and closed circles are identified with pGADD2-2, whereas none of the tumors are showing BLI with pGADD1-1. Experimental details in Materials and Methods.

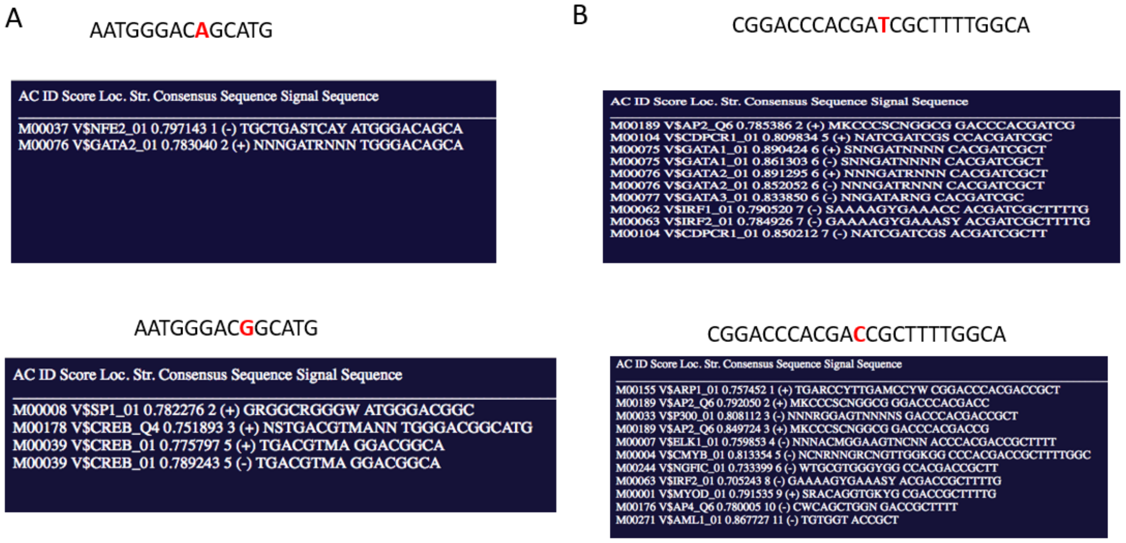

Figure S3. Transcription factors involved in cancer specificity. (**A**) A short sequence which had mutation (with A to G) was used to scan for transcription factors using online bioinformatics tool and resulting transcription factors are shown. (**B**) A short sequence which had mutation (with T to C) was used to scan for transcription factors using online bioinformatics tool and resulting transcription factors are shown.

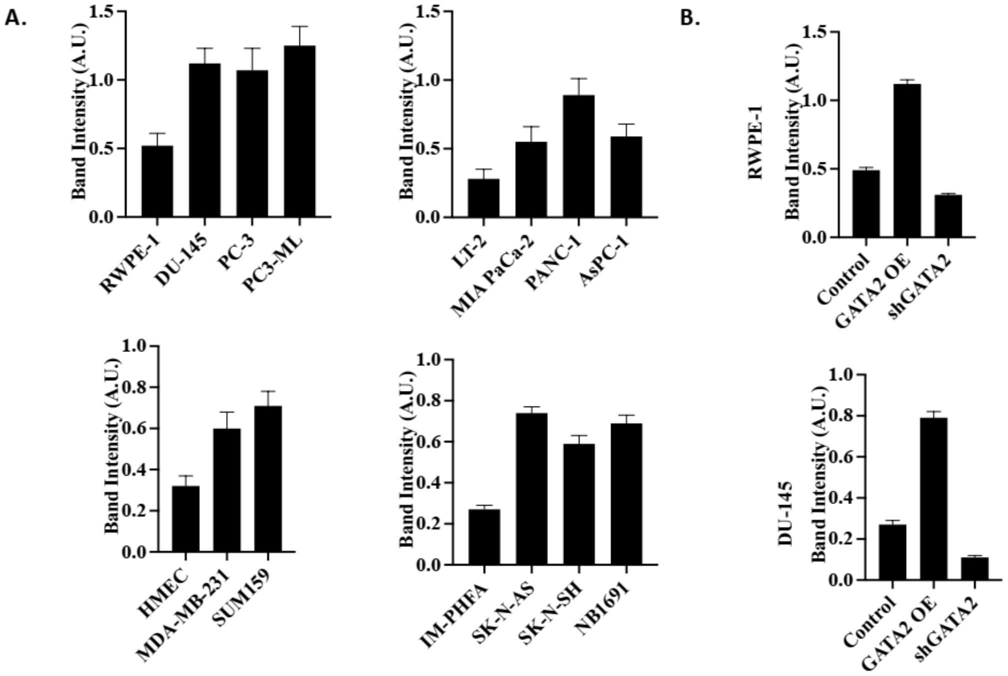

Figure S4. Densitometry analysis of Western blots from Figure 4.

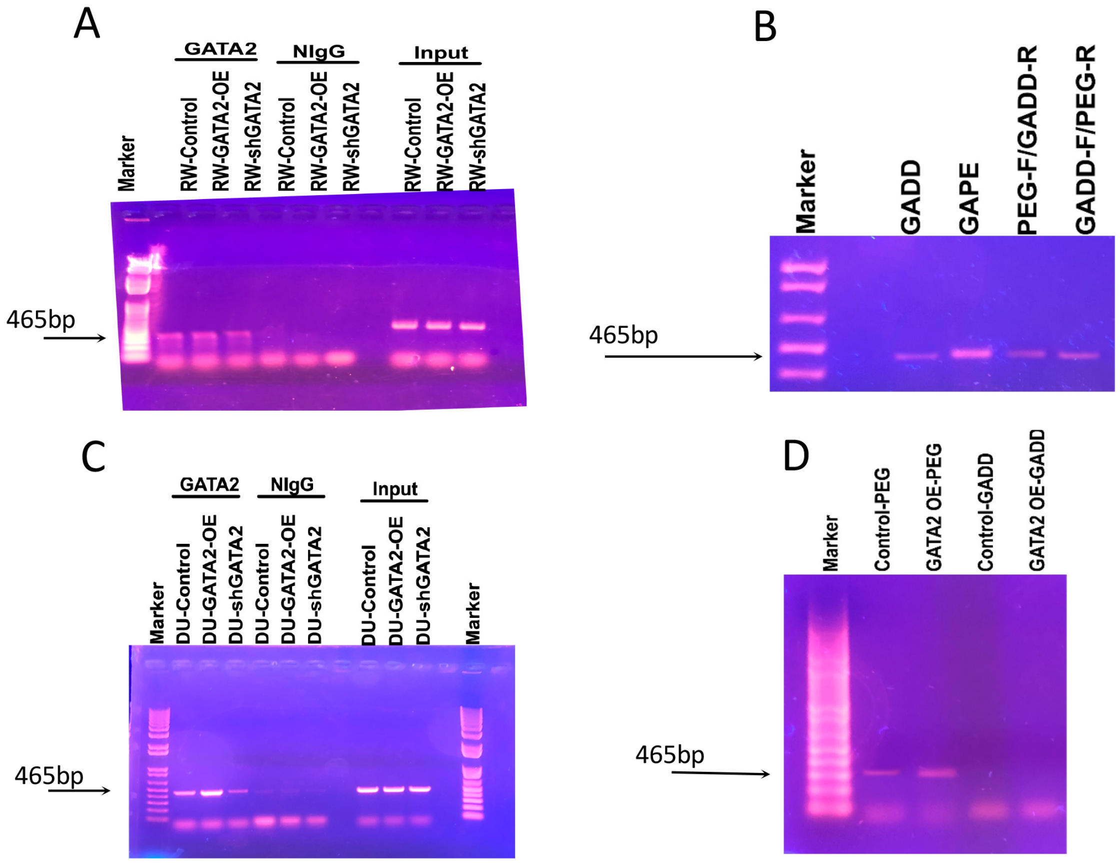

Figure S5. (**A**) GATA2 was either overexpressed or downregulated in RWPE-1 cells, ChIP assays were performed using GATA2 antibody and using GAPE primer sets with pGAPE as the target for PCR amplification. (**B**) DU-145 cells were transfected with pGAPE (GAPE-Prom) for 48 hours and used for CHIP assays, ChIP assays were performed using the GATA2 antibody and using different primer sets (pGAPE, pGADD, pGADD1-1 (PEG-F/GADD-R) and pGADD2-2 (GADD-F/PEG-R). (**C**) GATA2 was either overexpressed or downregulated in DU-145 cells ChIP assays were performed using the GATA2 antibody and using GAPE primer sets with pGAPE as the target for PCR amplification. (**D**) DU-145 cells were transfected with pGAPE (pPEG) for 48 hours and used for CHIP assay, ChIP assays were performed using the GATA2 antibody and using different primer sets (GAPE primer set and GADD primer set).

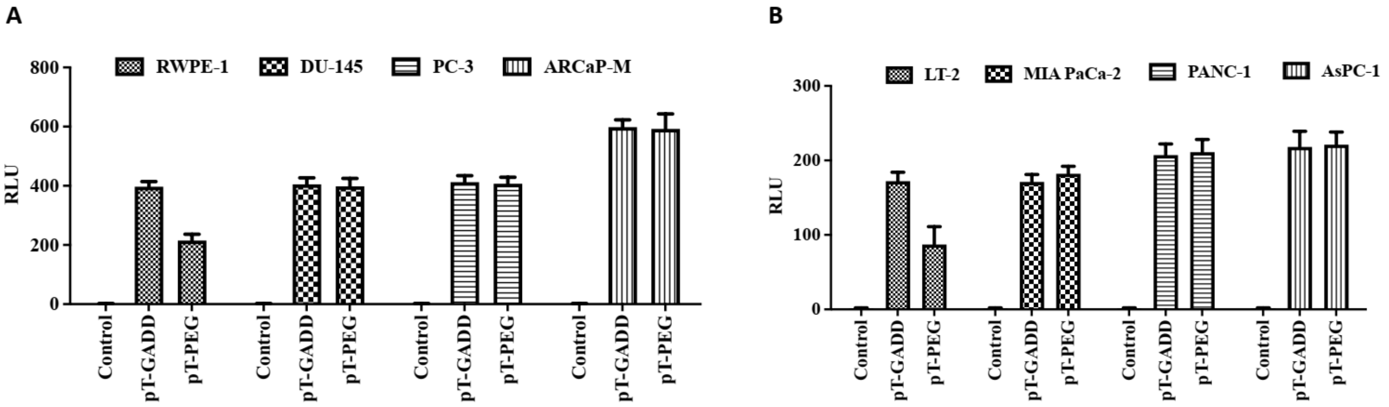

Figure S6. Activity of full-length GADD and PEG promoter in normal and cancer cells: (**A**) Immortalized human. prostate epithelial (RWPE-1) and prostate cancer (DU-145, PC-3 and ARCaP-M) cells (**B**) Human immortalized pancreatic mesenchymal (LT-2) and pancreatic cancer (MIA PaCa-2, PANC-1 and AsPC-1) cells were transfected with PGL4-Luc (Control), pT-GADD, pT-PEG.

Expression was normalized using pRL-TK, and the luminescence readings were plotted as relative luminescence units (RLU). The results presented are from three independent experiments.

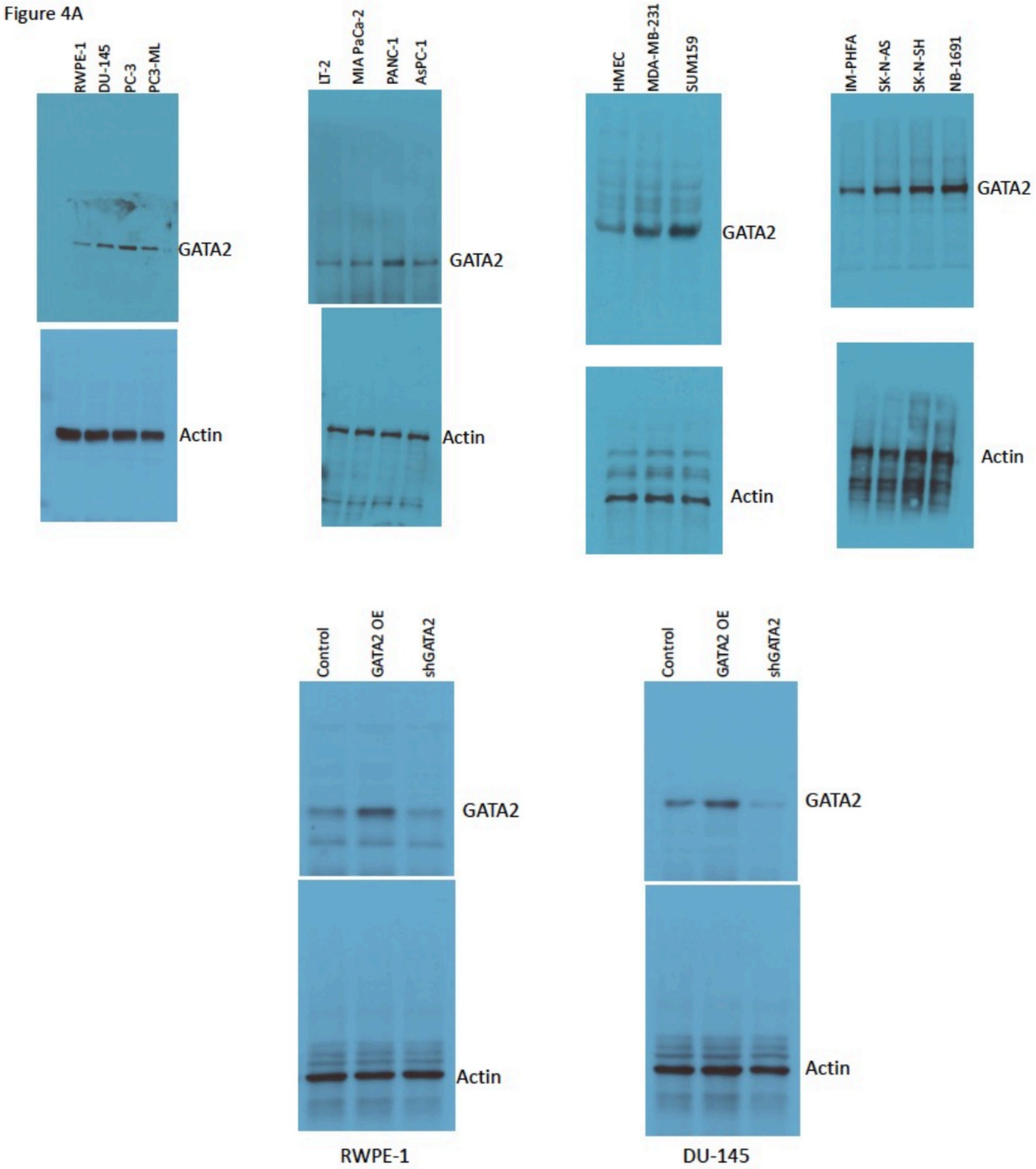

Figure S7. Original western blots for Figure 4.
